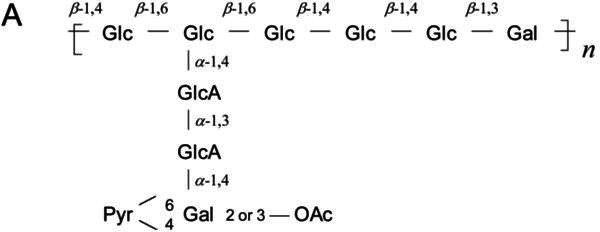# Correction for Staehelin et al., “Exo-Oligosaccharides of *Rhizobium* sp. Strain NGR234 Are Required for Symbiosis with Various Legumes”

**DOI:** 10.1128/jb.00157-25

**Published:** 2025-08-20

**Authors:** Christian Staehelin, Lennart S. Forsberg, Wim D'Haeze, Mu-Yun Gao, Russell W. Carlson, Zhi-Ping Xie, Brett J. Pellock, Kathryn M. Jones, Graham C. Walker, Wolfgang R. Streit, William J. Broughton

## AUTHOR CORRECTION

Volume 188, no. 17, p. 6168-6178, 2006, https://doi.org/10.1128/jb.00365-06. Page 6169: Fig. 1A should appear as shown in this correction. We have noticed that the chemical structure of the repeating subunit of the acidic EPS from strain NGR234 was incorrectly copied from the original paper (Djordjevic et al., 1986, Carbohydr Res 148:87-99). In the EPS of NGR234, both glucuronic acid residues adopt the α-anomeric configuration, as confirmed by Rodríguez-Navarro et al. 2014 [PLoS One 9(12): e115391]. This correction does not affect any conclusions drawn in our article. We apologize for any confusion or inconvenience this error may have caused.

**Fig 1:**